# Extent of Linkage Disequilibrium in the Domestic Cat, *Felis silvestris catus,* and Its Breeds

**DOI:** 10.1371/journal.pone.0053537

**Published:** 2013-01-07

**Authors:** Hasan Alhaddad, Razib Khan, Robert A. Grahn, Barbara Gandolfi, James C. Mullikin, Shelley A. Cole, Timothy J. Gruffydd-Jones, Jens Häggström, Hannes Lohi, Maria Longeri, Leslie A. Lyons

**Affiliations:** 1 Department of Population Health and Reproduction, School of Veterinary Medicine, University of California Davis, Davis, California, United States of America; 2 Genome Technology Branch, National Human Genome Research Institute, National Institutes of Health, Bethesda, Maryland, United States of America; 3 Department of Genetics, Texas Biomedical Research Institute, San Antonio, Texas, United States of America; 4 The Feline Centre, School of Veterinary Science, University of Bristol, Langford, Bristol, United Kingdom; 5 Department of Clinical Sciences, Swedish University of Agricultural Sciences, Uppsala, Sweden; 6 Department of Veterinary Biosciences, Research Programs Unit, Molecular Medicine, University of Helsinki, and The Folkhälsan Research Center, Helsinki, Finland; 7 Dipartimento di Scienze Veterinarie e Sanità Pubblica, Università di Milano, Milano, Italy; University of Uppsala, Sweden

## Abstract

Domestic cats have a unique breeding history and can be used as models for human hereditary and infectious diseases. In the current era of genome-wide association studies, insights regarding linkage disequilibrium (LD) are essential for efficient association studies. The objective of this study is to investigate the extent of LD in the domestic cat, *Felis silvestris catus,* particularly within its breeds. A custom illumina GoldenGate Assay consisting of 1536 single nucleotide polymorphisms (SNPs) equally divided over ten 1 Mb chromosomal regions was developed, and genotyped across 18 globally recognized cat breeds and two distinct random bred populations. The pair-wise LD descriptive measure (*r* ^2^) was calculated between the SNPs in each region and within each population independently. LD decay was estimated by determining the non-linear least-squares of all pair-wise estimates as a function of distance using established models. The point of 50% decay of *r^2^* was used to compare the extent of LD between breeds. The longest extent of LD was observed in the Burmese breed, where the distance at which *r^2^* ≈ 0.25 was ∼380 kb, comparable to several horse and dog breeds. The shortest extent of LD was found in the Siberian breed, with an *r^2^* ≈ 0.25 at approximately 17 kb, comparable to random bred cats and human populations. A comprehensive haplotype analysis was also conducted. The haplotype structure of each region within each breed mirrored the LD estimates. The LD of cat breeds largely reflects the breeds’ population history and breeding strategies. Understanding LD in diverse populations will contribute to an efficient use of the newly developed SNP array for the cat in the design of genome-wide association studies, as well as to the interpretation of results for the fine mapping of disease and phenotypic traits.

## Introduction

A variety of agricultural species was domesticated during the Neolithic revolution, over the course of which specialized breeds emerged. However, the domestic cat, *Felis silvestris catus*, escaped active and intentional breed development until the 19^th^ century [1], [2]. Cat domestication is hypothesized to have originated in the Near East from a still existing wildcat progenitor, *Felis silvestris lybica* ssp. [3], [4]. The archaeological record suggests that cats had a symbiotic relationship with humans, probably as vermin control, and potentially as part of ancient rituals [5]. The newly tamed cats roamed freely around human settlements, randomly breeding as a feral population and occasionally intermingling with the wild progenitor populations [6], [7]. As cats entered into a symbiotic relationship with humans, they altered their wild behavior patterns towards that of more tamed companions. After the initial behavior change associated with domestication, most cats have been selected by man mainly for simple aesthetic traits [2], rather than complex behaviors and qualities, such as hunting skills, speed, horsepower, and agriculturally important traits.

The development of cat breeds has followed four major strategies. The primary and historically most common strategy has been the manipulation of a subset of cats from a natural population. Persian, Turkish Angora, Maine Coon, Norwegian Forest Cat, and Siberian breeds are each likely to have arisen from specific feral random bred cat populations that were segregating for the longhair mutations [8], [9], [10]. The second strategy involves selection on a novel mutation that produces a unique trait that arose in a single cat, generally also from a random bred population. Breeders used selective mating to fix these traits to define the breed. The rexoid mutation of the Devon Rex, approximately 50 years old, represents one of the earliest novelty mutations found in random bred cats of the United Kingdom [2], [11], [12]. A more recent example of the second strategy is the Selkirk Rex breed, which is a curly coated breed with a single founding curly-coated cat identified in the USA [13]. The third path for cat breed development is to mix lineages, thereby creating cross-breed hybrids, such as the Ocicat or the Burmilla. Finally, some cat breeds are interspecies crosses. One example is the Bengal, which is a popular hybrid between domestic pedigree cats, such as Abyssinians and Egyptian Maus, and Asian Leopard cats, *Prionailurus bengalensis* [14]. Because of the recent developments in cat breeding, the focus on Mendelian or quasi-Mendelian traits, and variation in breed development strategies, one expects different levels of Linkage disequilibrium (LD) across the breeds.

Linkage disequilibrium (LD) is the non-random association of alleles at different loci on chromosomes in a gamete. A *priori* knowledge of the extent of LD has proven to be instrumental in the design of GWA studies and fine-mapping of genetic diseases [15]. LD analyses have been determined in domesticated animals, such as cattle [16], sheep [17], [18], and pig breeds [19], [20]. For companion animals, detailed analyses of wild and domestic canids shows that LD of dog breeds extends from a few hundred kilobases to megabases, with great variation between breeds [21], [22], [23], [24]. LD has also been estimated in horses using selected regions and genome-wide comparisons [25], [26]. The prevalence of LD in the horse is less pervasive than in the dog, and little variation is detected between breeds. Estimates of LD can provide predictive value for the design and implementation of GWA studies in the cat using the IIlumina array (Infinium Feline 63K DNA iSelect). This will facilitate understanding of breed-related genetic abnormalities as models for human diseases.

The alternative strategies to develop cat breeds create inherent difficulties in estimating LD. The natural decay of LD via recombination and gene conversion is suppressed in the development of domesticated breeds. LD within breeds is maintained by population genetic forces, such as selection, variation in migration substructure, bottlenecks, and inbreeding [27], [28]. LD in cat breeds that have been recently established from the feral population may be low, but the intensity of these pressures may have increased LD from the ancestral baseline. To evaluate the cat breeding dynamics and provide important estimates for GWA studies, the LD of 18 cat breeds and two diverse random bred populations was examined. Approximately 1500 SNPs covering ten regions of 1 Mb were genotyped in 408 cats. The identified LD should assist in the efficient use of SNP arrays for association studies and facilitate fine-mapping strategies in cat genomic studies.

## Materials and Methods

### Ethics Statement

Pedigreed and random bred cats samples, used in this study, were collected during the period 1994–2011 and stored as archival of DNA samples. The samples were collected by laboratory personnel at cat shows and field trips, or sent by collaborators, breeders, and cat owners from various countries. All samples were collected via buccal (cheek) swabs with the exception of those from collaborators including Birman, Burmese (F), Maine Coon, and Norwegian Forest Cat, which were collected as whole blood. The samples used in this study were selected from the DNA archive and meet selection criteria discussed below.

### Sample Collection and Preparation

Eighteen globally recognized cat breeds, 8–20 cats per breed, were selected based on their worldwide popularity, USA population size, population genetic distinctiveness, and breed history [1], [29], [30] (Table 1). Breed individuals were pedigree-verified to be unrelated at least to the grandparent level. The Burmese, Korat, and Turkish Van were available as both domestic and foreign cats to examine differences in breed development in different countries. Breeds were also selected based on breed development strategy and popularity. The Ocicat represents a breed that was formed by purposely hybridizing different breeds. Older, well-established breeds were included for analyses, such as the Abyssinian, Persian, and Manx, as well as younger breeds, including the Siberian and Egyptian Mau. The Cornish Rex is selected and fixed for a breed defining trait, while Russian Blues are accepted in only a blue color. Both breeds are among the less popular cat breeds.

**Table 1:** Population and statistic summary of cat breeds for LD study.

Breed/Random bred	Abr.	(n)	% SNPsincluded	50% decay		% decay inflation MAF ≥0.1*	Fraction SNPs at 40–60 Kb**
				*r^2^*	Dist (Kb)		
Abyssinian	ABY	21	65.3	0.24	96	52.0	0.05
Turkish Angora	ANG	14	67.9	0.25	29	29.3	0.02
Birman	BIR	20	67.2	0.25	186	52.3	0.10
Burmese (Domestic)	BURD	19	50.2	0.25	380	34.8	0.19
Burmese (Foreign)	BURF	19	38.6	0.25	249	43.8	0.07
Chartreux	CHA	8	63.7	0.27	66	39.4	0.09
Cornish Rex	COR	21	69.4	0.24	63	48.4	0.04
Egyptian Mau	EGY	9	67.5	0.27	87	61.8	0.12
Japanese Bobtail	JAP	12	69.7	0.26	37	22.9	0.02
Korat (Domestic)	KORD	19	51.9	0.25	75	32.4	0.05
Korat (Foreign)	KORF	17	48.5	0.25	101	48.2	0.08
Maine Coon	MAIN	19	72.6	0.25	154	50.2	0.14
Manx	MANX	20	88.9	0.24	25	3.8	0.03
Norwegian Forest Cat	NFC	20	72.2	0.25	68	43.3	0.13
Ocicat	OCI	21	66.1	0.24	148	46.0	0.08
Persian	PER	19	66.3	0.25	74	35.1	0.10
Russian Blue	RUS	18	66.6	0.25	43	50.0	0.04
Siamese	SIA	19	56.9	0.25	230	39.8	0.13
Siberian	SIB	19	78.8	0.25	17	32.0	0.03
Turkish Van (Domestic)	VAND	19	75.2	0.25	44	48.8	0.04
Turkish Van (Foreign)	VANF	12	53.6	0.26	67	58.1	0.05
Random Bred (Eastern)	ERB	22	61.2	0.24	36	26.5	0.03
Random Bred (Western)	WRB	21	84.2	0.24	19	38.7	0.03
Random Bred (Combined)	RB	***	81.3	0.23	18	37.9	0.02
	Avg.	18	66.0	0.25	96	40.7	0.07
	Total	408					

* Percent inflation in the estimate of the extent of LD when using MAF ≥0.1.

** Fraction of pairs of SNPs at distance class (40–60 Kb) with *r^2^* value ≥0.8.

*** Random bred is combing both eastern and western random bred samples.

Two random bred populations were selected based on their genetic distinctness and geographical isolation [4], [31]. One random bred population represents East Asian random bred populations, selected from Chinese feral cats. The Western random bred population is represented by non-breed household pets presented to veterinary clinics in Hamburg, Germany. Random bred cats were assumed to be unrelated. As a representation of an overall random bred population, the two random bred populations were combined as one population (RB). The DNA samples of random bred cats were available from previous studies [31]. Ninety-seven DNA samples were genotyped directly and 311 samples were whole-genome amplified (WGA) using Qiagen Repli-g Mini Kits (Qiagen Inc., Valenica, CA, USA) to obtain the preferred concentration of at least 20 ng/μl for the SNP genotyping assay.

### SNP Array Development

SNPs (N = 1536) were selected over ten different 1 Mb chromosomal regions (Chrs. A1, A2, B3, C2, D1, D2, D4, E2, F2, and X) (Table S1). The chromosomal regions were selected based on (i) sampling from regions at various positions in relation to centromere, mid-arm, and telomere of chromosomes that varied in size (Figure S1a), (ii) contiguous map in comparison to the dog genome, (iii) containing long contigs in the cat genome assembly, and (iv) having good SNP coverage and representation. The regions’ genomic features such as GC content, number of genes, and number of simple repeat elements were obtained from UCSC Genome Bioinformatics website (http://genome.ucsc.edu/), using algorithms therein. Approximately 150 SNPs were distributed over each 1 Mb region of each chromosome with varying spacing (Figure S1b). SNP density was intentionally increased in the first 100 Kb at one end of each of 1 Mb region to allow fine-scale estimation of LD. The selected SNPs were pooled from a mix of the SNP discovery individuals; (i) cat genome sequenced at 1.9× coverage – Abyssinian [32], (ii) American shorthair, Cornish Rex, European Burmese, Persian, Siamese, Ragdoll [33], (iii) 9–13× sequencing of five individuals each of Birman, Japanese Bobtail, Norwegian Forest Cat, Maine Coon, Turkish Van, Egyptian Mau, and East Asian random bred (Table S2). SNPs that scored high in illumina inclusion design and were polymorphic in at least two breeds were placed on the custom array (Table S2).

SNP genotyping was performed at the Texas Biomedical Research Institute (San Antonio, TX). Array data was analyzed using the illumina GenomeStudio software (version 1.7.4) to obtain genotypes. SNP genotype clusters were manually evaluated and SNPs that (i) failed genotyping, (ii) were poorly clustered, or (iii) were monomorphic, were removed from downstream analysis (Table S1).

### Population Analyses

To ascertain F^st^ across breeds, a supervised analysis using *ADMIXTURE* 1.22 was conducted, with each of the 23 populations set as a specific K [34]. The process was repeated 30 times with a pseudo-random seed (computer time) and the mean pair-wise distance values across populations were computed from the matrices. The resultant F^st^ matrix was visualized using R in the form of an unrooted neighbor-joining tree as per McEvoy et al. [35] with the *APE* package and the Neighbor-Joining Tree Estimation function (*nj*) based on methods of Saitou and Nei [36], [37].

Using the total number of SNPs (n = 1463), observed and expected heterozygosities, and F_is_ were calculated for each population in each of the ten chromosomal regions. Tajima’s D was calculated using the *pegas* package in R [38].

### Linkage Disequilibrium Analysis

SNP genotype analysis and LD pair-wise calculations were performed using the *genetics* package in R [39]. SNPs were analyzed separately in each population to remove SNPs with a call rate <80% and those that were monomorphic within a population. To account for sensitivity of LD measures to variance of allele frequencies [40], [41] and effect of minor allele frequency (MAF) on the summary of the extent of LD, two MAF, 0.1 and 0.05, were evaluated for each population, separately [42].

The squared correlation coefficient (*r^2^*) was calculated between each pair of SNPs on the same chromosome [43]. The background LD was estimated for each population by averaging all pair-wise estimates between all SNPs on different chromosomes using MAF ≥0.1. A general representation of the extent of LD in each population and a comparison between populations was estimated using the decay of the LD measures, *r^2^* for each chromosome, separately, and as a summary of the combined data of autosomal chromosomes (denoted Auto) and all chromosomes (denoted All).

The decay of the *r^2^* was estimated by approximating the least squares fit line using the *nls* function in R [44]. The nonlinear least-squares of *r^2^* estimates was approximated using a model of the expected value of *r^2^* under drift-recombination equilibrium [45] that has previously been implemented [46], [47].

This approximated decay line was performed separately in each population for each of the ten chromosomes, the autosomal chromosomes combined, and all chromosomes combined. Due to variation in the rate of decay of the *r^2^*, the modeled decay of *r^2^* was plotted as a function of distance in kilobases and the decay point where *r^2^* reaches 50% of its maximum value was chosen as the comparison point between populations. To evaluate and predict the usefulness of the newly developed array for GWA studies in each population, the fraction of SNP pairs with *r^2^* value ≥0.8 were estimated in different inter-SNPs distance classes.

### Haplotype Analysis

Haplotype analysis was performed using Haploview 4.2 [48]. Haplotype blocks were defined for each of the 1 Mb chromosomal regions separately for each population using the Solid Spine of LD option with a MAF ≥0.05 and all other parameter set to default. Haplotype diversity was measured by total number of haplotype blocks defined and total haplotypes observed in each chromosomal region and collectively for the ten regions. Visualization of haplotype blocks and haplotype frequencies therein were visualized in R.

## Results

### Cats and SNP Analysis

Well-recognized breeds with a comprehensive historical and demographic record were selected for analysis (Table 1). Samples of direct DNA source were 23.8% (n = 97) whereas 76.2% were WGA (n = 311) samples. The samples of direct DNA source were under-represented in each population (Table S3). To examine if WGA samples experienced allele dropout and overall reduced heterozygous genotypes among its individuals, the heterozygous genotypes were counted in each individual of populations with representation of the two DNA sources. The differences in the number of heterozygous genotypes between the two groups did not reach significance in most of the populations when a t-test was applied (Table S3). The Manx breed, which has a near equal representation of the two sample sources, showed no significant differences (*p* = 0.77). The Korat (D) and Western random bred populations attained statistical significance on this measure. However, the inadequate sample size for these two populations warrants caution against accepting the t-test results as definitive, and requires further investigation. Control replicates (n = 6) showed no allele-calling inconsistencies and a per SNP no call rate of 0.005%.

**Figure 1:**
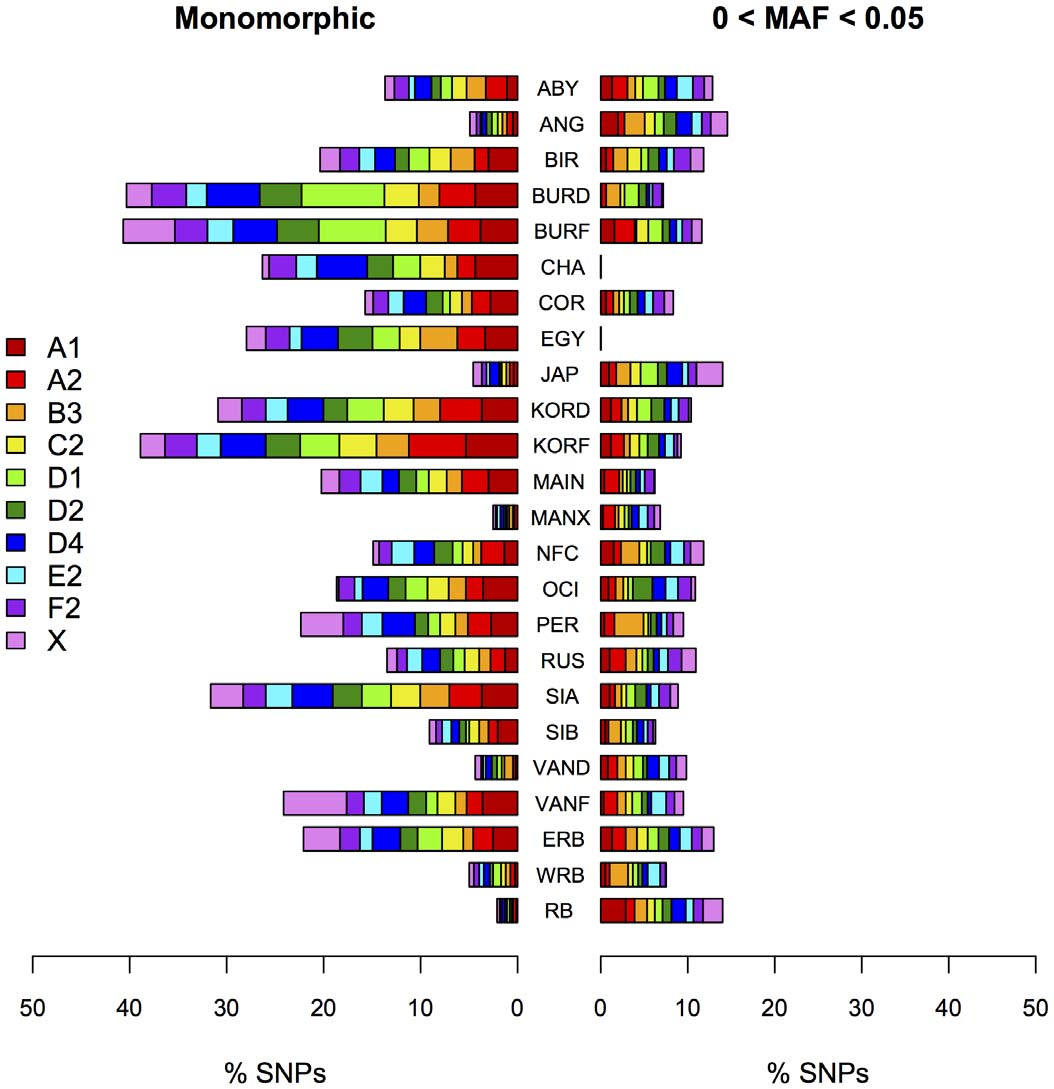
Population based SNP analysis of the domestic cat. Number of monomorphic SNPs (left) and SNPs with a minor allele frequency 0< MAF <0.05 (right). Legend corresponds to the ten chromosomal regions.

All cats analyzed in the study have genotype call rates above 75% for the SNP data. Initial genotype cluster analysis of the 1536 SNPs that passed design showed that 73 SNPs (5%) failed in the assay (Table S1). Overall, 1463 (95%) SNPs were analyzed in each population separately and an average of 5.7% of the SNPs had call rates <80% per population, ranging between 0.6% and 12.8% (Table S3). Within populations and breeds, the average number of monomorphic SNPs was 19%, ranging between 2.1–40.7%. (Figure 1, Table S3). An average of 9.4% of the SNPs had a MAF <between 0 and 0.05 across all populations and ranging between 0% and 14.6% (Figure 1, Table S3). Overall, after excluding monomorphic, MAF <0.05, and call rate <80% SNPs from each population independently, an average of 66% (n = 965) of the SNPs were included in downstream analysis, ranging from 38.6% in the foreign Burmese population to 88.9% in the Manx breed (Table 1).

### Population Analysis

**Figure 2:**
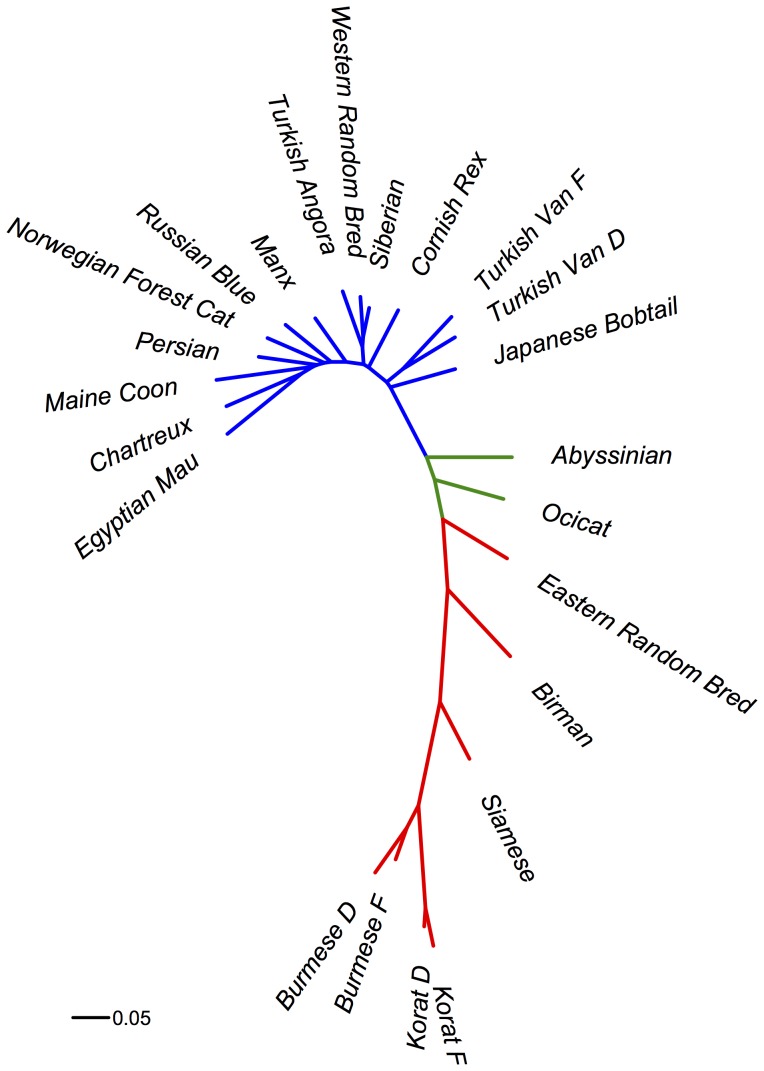
Domestic cat population analysis. F_st_ based neighbor-joining tree of cat populations. Red color represents eastern derived populations, blue represents western derived populations, and green color represents other populations.

The populations’ phylogenetic relationship was studied by examining pair-wise F_st_. The neighbor-joining tree illustrated a clear separation between eastern and western populations (Figure 2). Western breeds appeared less distant from each other (Figure 2 - colored blue) when compared to eastern breeds (Figure 2 - colored red). The scaling of the branches of the neighbor-joining tree of the western breed reflects the relatively recent developmental history of the breeds. These lineages were subject to artificial selection and went through bottlenecks relatively recently, therefore experiencing less drift divergence from the most recent common ancestor. Additionally, the close relationship between the breeds supports the reported introgression between populations during the breeds’ development. In contrast, the eastern breeds exhibit longer branch lengths due to higher F_st_ values, which reflect demographic history. These breeds historically attest to have had older population divergences and more defined breed structures when compared to the western counter parts. Therefore there has been more time for divergence due to drift and separation to increase genetic distance values. Finally, the Abyssinian and Ocicat breeds appeared equidistant between eastern and western populations. The Abyssinian’s breed history is not fully known and many speculate independent breed development in Africa. Therefore, Abyssinians may be neither eastern nor western. In contrast, the Ocicat is a breed developed as a hybrid of Abyssinian and Siamese cats, explaining the shift towards the eastern populations. Sister populations (Burmese, Korat, and Turkish Van) exhibited low genetic distance values, as expected. The Japanese Bobtail appeared more closely related to western populations than to eastern populations.

Observed and expected heterozygosities varied across populations, with mean values of 0.251 and 0.242, respectively. Similarly, the mean inbreeding coefficient ranged between −0.055 and −0.012 (Table S3). Tajima’s D was estimated for each population on each region separately (Figure S2a). The mean estimated Tajima’s D across chromosomes for each breed ranged between −3.6 and 0.31. With the exception of the Norwegian Forest Cat breed, all populations exhibited mean Tajima’s D between −1.17 and −3.6 in accordance with the presumed population expansion that took place following the breeds’ formation bottlenecks (Table S2, FigureS2b). The mean Tajima’s D estimates across populations for each chromosomal region revealed no pronounced differences between chromosomal regions (Figure S2c).

### Linkage Disequilibrium

The effect of MAF cut-off choice on the extents of LD was evaluated by estimated LD using two MAF, 0.05 and 0.1. Variation resulting from choice of MAF was apparent (Figure S3a). The Manx breed showed small differences in the decay of LD and the estimates of the extent of LD, whereas all other populations exhibited extreme differences. The magnitude of differences was independent of sample size (Figure S3b–d). The inflation ranged between 3.8–61.8% (Table 1). Using a MAF of 0.1 inflated the estimates of the extent of LD by an average of 40.7%, illustrating how a slight difference in MAF choice could have significant differences in the estimate of the extent of LD. Therefore, a MAF of 0.05 was chosen for further detailed analyses and comparisons.

**Figure 3:**
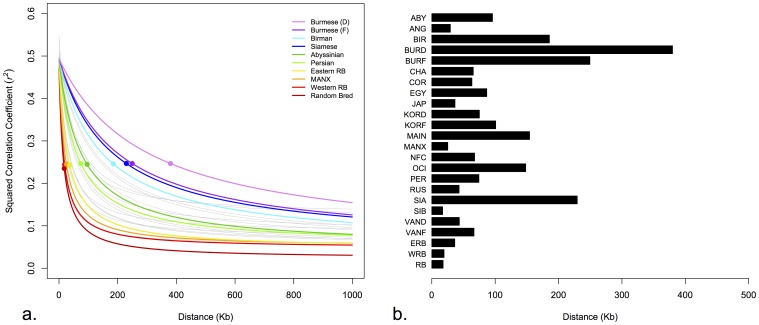
Extent of LD in domestic cats. a) Decay of *r^2^* value of autosomal chromosomes combined as a function of distance (Kb). Colored decay lines represent selected populations that depict upper, middle, and lower estimates of the LD measures. Gray decay lines represent the remaining populations. Filled circles indicate the point of 50% decay of *r^2^* maximum value. b) Distance in kilobases at which *r^2^* decays to 50% of the maximum value, which is the distance at *r^2^* ≈ 0.25. The distances correspond to the filled circles in (a).

Decay of *r^2^* value was analyzed for each chromosome separately and for combined chromosomes. The chromosome specific LD decay varied among populations but the relative order of population decay rate was not consistent across chromosomes (Figure S4, Table S4). The decay of the *r^2^* value of combined autosomal chromosomes was chosen to avoid a biased comparison that might result from insufficient numbers of pair-wise comparisons and elevated levels of LD on the X chromosome (Figure 3a). The point of 50% decay *r^2^* value extended to a range of distances that was as low as 17 kb in the Siberian breed and as high as 380 kb in the domestic Burmese breed (Figure 3b). The mean extent of LD across all populations was 96 kb (Table 1). The greatest amount of LD was found in the eastern breeds; Burmese (D,F), Siamese, and Birman. Western breeds exhibited intermediate levels of LD, with maximal levels in the Maine Coon. Overall, 14 out of the 21 breed populations exhibited an extent of LD <100 kb (Table 1, Figure 3).

LD was assessed for all SNPs across the different chromosomal regions to estimate a background level of LD (Table S5). Using a MAF >0.1 would not have a significant effect on the mean estimate of the background LD because a large number of pair-wise comparisons were performed, and these data were not used to summarize LD of a region. Background LD ranged from 0.05 in the Eastern random bred population to 0.11 in Japanese Bobtail, with an across population average of 0.07. The background LD was 0.04 when the Eastern and Western random bred populations were combined. Turkish Angora, Chartreux, Japanese Bobtail, Manx, combined random bred cats, Siberian, and Western random bred cats approach the background *r^2^* decay level at 1 Mb distance. All other cat populations do not approach the background level of LD.

**Figure 4:**
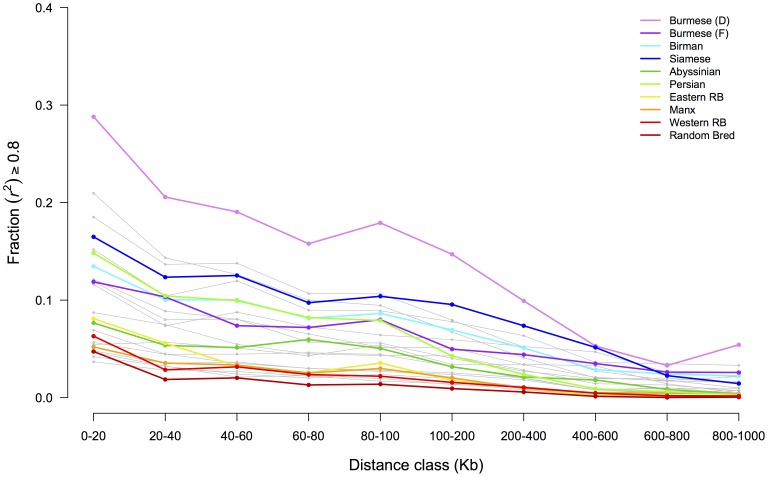
Fraction of GWAS-informative SNP pairs at different distance classes in domestic cat breeds. Colored lines represent selected populations that depict upper, middle, and lower examples whereas the gray lines represent the remaining populations (Table S6).

The fraction of SNP pairs with *r^2^*≥0.8 was investigated in each population over different distance classes, which represent the range of SNPs informative for GWAS (Figure 4, Table S6). The 40–60 Kb distance class generally represents the density of the newly developed SNP array for cats and thus was used to compare across populations. Domestic Burmese were found to have the highest fraction of GWAS-informative SNPs, over 19%. Birman, Egyptian Mau, Maine Coon, Norwegian Forest Cat, Ocicat, Persian, and Siamese exhibited fractions slightly greater than 10%. The remainder of the populations had less than 10% GWAS-informative SNPs (Table 1).

### Inter-chromosomal LD Comparison

To determine if significant inter-chromosomal variations in LD decay were present in the cat, the LD decay was determined for each chromosome in each population. The extent of LD in all ten chromosomal regions separately and combined were summarized (Figure S4 and TableS4). Three chromosomes (A1, B3, and X) displayed a relatively long extent of LD, where the decay of 50% of *r^2^* was achieved on average at over 300 kb, while the C2 and E2 exhibited significantly shorter extent of LD (Figure S4). The extent of LD did not appear to correlate with the current estimated size of the chromosomes (*p* = 0.579). The LD on the X chromosome extended further than any autosome. Using Pearson’s test of correlation between the mean extent of LD across populations and the molecular genomic features of each region, no correlation has been found between the extent of LD in a region and the GC content (*p* = 0.63), number of gene (*p = *0.28), or number of simple repeat elements (*p* = 0.21).

### Haplotype Analysis

A comprehensive haplotype analysis was conducted for the populations under study. The haplotype blocks and the number of haplotypes within each block were compared across all populations in each chromosomal region (Figure S5). The length of the haplotype blocks within each region mirrored that extent of LD (Figure S4, Figure S5). The chromosomal regions in A1, B3 and X showed extended haplotype blocks across all populations whereas chromosomal regions C2 and F2 showed less extended blocks.

**Figure 5:**
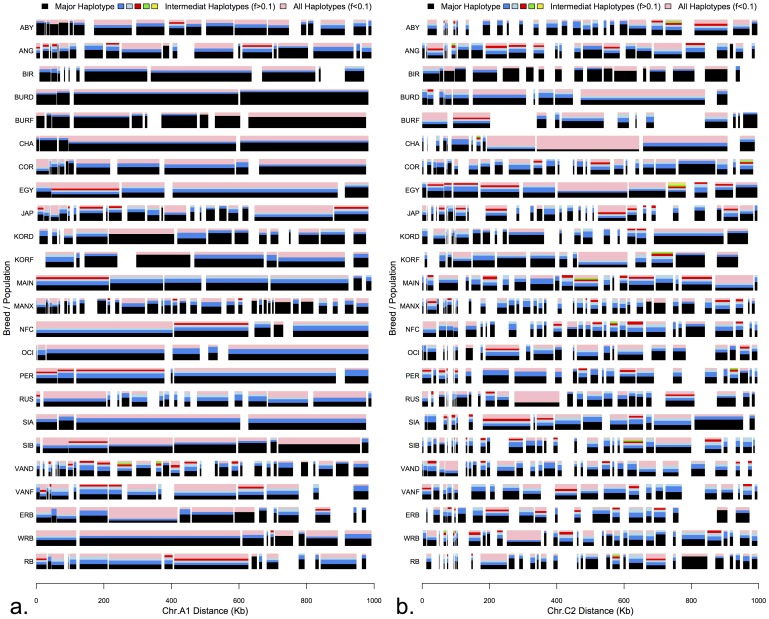
Domestic cat haplotype structure and diversity of two autosomal regions. a) Haplotype analysis of chromosome A1 region, which exhibits long extent of LD. b) Haplotype analysis of chromosome C2 region, which exhibits short extent of LD. Breeds/populations are represented on the y-axis and the position of haplotype blocks are shown on the x axis. Haplotype blocks are represented by horizontal rectangles. The frequency of individual haplotypes within a block is proportional to the height of the color. Major haplotype is represented by black color, intermediate haplotypes with frequency >0.1 are represented by blue, light blue, red, green and yellow, respectively. Pink color represents the sum of the frequencies of all haploypes with a frequency <0.1. Empty regions between blocks indicate lack any informative markers (MAF ≥0.05) and can be considered monomorphic across all individuals within a population.

As an example, chromosomal region A1 and C2 were chosen for a detailed comparison across breeds (Figure 5). In region A1, the eastern breeds, Birman, Burmese (D,F), Korat (D,F), and Siamese exhibited longer and fewer haplotype blocks, each with a major haplotype dominating each block. Their western counterparts exhibited shorter blocks with greater variation in the number of haplotypes within each block (Figure 5a). Similarly, in region C2, while more haplotype blocks were found across all populations, the differences between eastern and western populations remained as in region A1 (Figure 5b).

As a measure of haplotype diversity, the total number of haplotype blocks and the total number of haplotypes in each region was compared across populations (Figure S6). The mean number of blocks across populations was ∼18, and ranged from 8.8–33.4, whereas the mean number of haplotypes was ∼103.5, ranging from 47.7–144.3 (Table S3).

## Discussion

Cat genetic resources such as linkage maps [49], [50], [51], radiation hybrid maps [52], [53], genetic markers [33], [54], and genome sequence [32], [33] have been useful for genetic mapping of diseases, but have significant limitations. The extended LD documented in other companion animals, such as dog and horse, has proven to be the key for successful genome-wide association studies, especially with relatively low-density DNA arrays [22], [55], [56], [57]. Understanding the extent of LD in cats would undoubtedly aid in performing effective genome-wide association studies. This study examined the population genetic statistics and phenomena, linkage disequilibrium and haplotype structure in domestic cat breeds. This study extended the understanding of cat breed population dynamic and assessed their utility in GWA studies.

### Cat and Companion Animals’ LD

In cat breeds, LD variation spans the range between horses and dogs, with no marked extremes. LD was shortest in the Siberian breed at ∼17 kb, and most extended in the domestic Burmese at ∼380 Kb. At a comparable *r^2^* value to the one used for the cat (*r^2^* ≈ 0.25), most horse breeds reach the same level of LD at <100 kb when using a similar study design [26], and in a genome-wide analysis [25]. The Thoroughbred, which has the highest LD among horse breeds, reached *r^2^* ≈ 0.25 at ∼400 kb [26] to 500 kb [58], which is comparable to the Burmese.

Similarly, in a study of LD in wild and domestic dogs, Gray et al. [21], reported and compared distances at which the *r^2^* value reached 0.2. The majority of dog breeds had LD extending geater than 200 kb, with only the Labrador Retriever and Saint Bernard reaching the same LD level as cats in <100 kb. The highest LD in dog breeds was found in the Mastiff, which extends over 5 Mb. Comparatively, the highest LD in cats was found in the domestic Burmese breed, extending approximately less than one tenth of the Mastiff LD distance.

### Population Histories of Cat Breeds

This study has focused on the most popular and genetically distinct cat breeds, many of which have several known inherited diseases [59], [60]. The selected breeds represent a range of population genetic parameters in terms of their demographic history; varied effective population size, diverse selection pressures of different magnitude, generation time since LD generating event (e.g., truncation selection or bottleneck), and ancestral genetic background. This variation enables exploration of the dependence of LD upon breed population dynamics and history.

The Burmese breed, both populations domestic (D) and foreign (F), displayed the largest extent of LD and the fewest and longest haplotype blocks among cat breeds. Although only a few cats were known to be the foundation of the breed, other cats were used for the breed expansion, such as Siamese. The Burmese quickly became one of the most popular breeds in the cat fancy, which contributed to a rapid population expansion. This population expansion is evident in Tajima’s D estimates for the breed, as it is in most breeds. A negative value, and in particular values below −2, are often indicative of either strong selective sweeps in a region, or rapid population expansion from a bottleneck. Because there was no discernible genomic variation in this statistic, it seems that one should assume that the values are being generated by genome-wide demographic processes. In particular, selection of a small number of founding individuals, and the rapid population growth through breeding, for some of these lineages, seems the most likely source of the recurrent high negative values across the populations.

The domestic Burmese population suffers from an autosomal recessive craniofacial defect [61], which has fractionated domestic Burmese into two non-cooperative groups, Traditional and Contemporaries. The presence of the craniofacial defect has caused many breeders to abandon the breed, resulting in a population crash. The foreign Burmese are known to have more recognized colors but suffer from diseases that are or appear to be familial, such as hypokalemia [62], [63], an orofacial pain syndrome [64], and diabetes [65]. Due to these population-specific genetic diseases, Burmese cat registries in the USA and abroad do not support exchange of cats and the two populations have been kept separate.

The large extent of LD, long haplotype blocks, deviated Tajima’s D, high levels of inbreeding, and reduced heterozygosity [4], [30] among Burmese cats are a testament to their specific demographic history as a population. Precisely, strong selection and a breeding program reliant on very few founders resulted in genomic signatures of low effective population size and inbreeding, such as high levels of LD and long haplotypes, which mark identity by descent tracts.

Four breeds, Siberian, Manx, Turkish Angora and Japanese Bobtail, had LD and haplotype structure comparable to random bred domestic cat populations. The Manx breed is defined by a single physical characteristic, the lack of a tail. The tailless trait is dominant and lethal *in utero* when homozygous, while heterozygotes have variable expression [66], [67]. To maintain the phenotype, despite natural and artificial selection against the genetic variant that produces it, tailless Manx are crossed with random bred cats. This is a viable path of breed characteristic maintenance because many color varieties are acceptable and a population of origin exists on the Isle of Mann, which can provide a readily available source for new founders and migrants. The recurrent introduction of migrants due to out-crossing and diversity of founders result in the increased genetic diversity of the Manx in relation to other breeds, explaining the relatively low level of LD and the similarity to a random bred set of cats.

Similarly, breeds such as Siberians, a new breed, and Turkish Angoras, tend to resemble the random bred street cats of their populations of origin, Russia and Turkey, respectively [31]. Both breeds have over a dozen allowable colors and pattern variations. The Japanese Bobtail appeared distant from population of origin in the east. This is due to re-establishment of the breed using various other breeds and populations, likely from the West [30]. Thus, as in the Manx breed, Japanese Bobtail low LD levels approach that of random bred populations, likely because these breeds resemble random bred populations in their population structure and history [31].

The Persian was one of the founding western-derived breeds for the cat fancy, and was presented at the first cat show in the United Kingdom in 1871 (though they were known as Angoras at the time). Historically, the most popular breed worldwide, Persian cats have a massive variety of colorations and patterns as well as the largest breeding population in the cat fancy [1]. The Persian’s LD is at an intermediate level in comparison to the other cat breeds, which is in agreement with the moderate levels of inbreeding and heterozygosity [4], [30]. These moderate LD levels might be the result of two opposing dynamics. First, the large effective population size likely tends to shorten the extent of LD. Because of the large number of breeding individuals from diverse backgrounds, the stochastic dynamics that normally might fix particular haplotype blocks rapidly and therefore increase the LD statistic are dampened. Conversely, strong and continuous selection for long hair and brachycephalic face increases LD by elevating the stochastic dynamics globally on a genomic scale by reducing effective population size and more notably locally due to selective sweeps around loci of interest.

Siamese is an ancient eastern-derived breed and also one of the foundation breeds for the cat fancy. This large and historically popular breed shows one of the highest levels of LD among the studied cat breeds. Acknowledging the large census population size of the breed, the high LD levels may be attributed to the breed pointed coat color trait that defines the breed [68], and the limited number of acceptable color variants. Both produce a divergence between census and effective population size due to strong selection and bottlenecks. Siamese cats have very strict breeding practices, mandating 12– generation pedigrees and prohibit out-crossing, necessarily reducing effective population size. An equally popular eastern breed is Birman, and it shows strikingly similar levels of LD to that of Siamese. The Birman breed census population is smaller than the Siamese, and is fixed for the pointed mutation and longhair with few recognized colorations. The levels of inbreeding and heterozygosity are near identical for these two breeds [4], [30], indicating concordance of effective population size due to bottlenecks and selection coefficients of similar magnitude.

Contrary to other breeds with fixed traits, both domestic and foreign Korat populations exhibit moderate LD levels. The expectation would be that the Korat would show high levels of LD due to its small census population size and single blue color presentation. The Korat is putatively an ancient lineage, but was only recently recognized as a breed in 1966. Although its census population size is small, breeders actively introduce random bred cats into their breeding stocks from the breed’s country of origin, Thailand, thereby reducing inbreeding depression and maintaining high levels of heterozygosity in accordance with previous genetic studies [4]. These population genetic indicators suggest therefore that the long-term effective population size of the Korat may be larger than that of Siamese or Birman, despite its small numbers as a breed.

### Inter-chromosomal LD

Inter-chromosomal LD variation was detected in the combined random bred population data set and in all populations. This pattern of variation likely reflects regional genomic variation in recombination rates, but exhibited no correlation with the size of the chromosome. Similarly, no correlation has been observed between the GC content of a region, gene number, or simple repeat number and the extent of LD. The X chromosomal region exhibited the highest extent of LD across all populations. This is attributed to the differences in effective population size of the X chromosome and the autosomes. The regional genomic variation suggests that the combined autosomal data is more representative of the breeds’ LD. Regional and chromosomal differences in recombination rates could be warrant future investigation.

### Extent of LD and Minor Allele Frequency

The MAF cut-off choice for the inclusion of SNPs in an LD analysis has profound effects on the results. Increasing the MAF from 0.05 to 0.1 inflated the estimate of the extent of LD by 40%. While this inflation in LD estimate was observed in the majority of the studied populations, Manx breed deviated from the trend and exhibited little inflation (Figure S3). This can be attributed to the number of SNPs included at both MAF cut-offs, 73% and 89% for MAF 0.1 and 0.05, respectively. An increase in MAF generally would result in an inflated LD estimate [42]. But, no inflation would be observed if enough pair-wise comparisons were calculated, suggesting that caution should be applied when estimating LD using a small number of markers or to interpret published results as in Sutter et al. [24].

### Cat and GWA Studies

Understanding the extent of LD in the domestic cat is important for the successful design and analysis of genome-wide association studies. The fraction of SNP pairs with *r^2^*≥0.8, which are considered GWAS - informative SNPs, allow the prediction of how successful GWA studies are likely to be in specific breeds. Domestic Burmese showed fractions of GWAS - useful SNPs ≥10% even in 200–400 Kb class distance. The eastern breeds, Burmese, Birman, and Siamese, show fractions of GWAS-informative SNPs ≥10% even at a class distance 100–200 Kb. At the class distance 40–60 Kb, which is equivalent to the density of the current 63K SNP array, Egyptian Mau, foreign Korat, Maine Coon, Norwegian Forest Cat, Ocicat, and Persian cats have GWAS-informative SNPs fraction ≥10%.

Eastern breeds are likely to generate successful GWA studies, particularly the domestic Burmese, where GWA studies are liable to be fruitful even with few cases and controls. Western breeds such as Turkish Angora, Cornish Rex, Japanese Bobtail, Manx, Russian Blue, Siberian, and random bred cats show smaller fractions of informative SNPs in distances equivalent to the current 63K array. GWA studies in such breeds may require larger numbers of cases and control or an array with higher density.

GWA studies focused upon breed defining phenotypic traits may be especially successful using the current DNA array. Positive selection pressure extends the LD from the locus of interest, producing an extended haplotype block around the causative genomic region. In such cases, SNPs that are at distance from the causative region might be in strong LD with the causal locus, signaling an association between the trait and that genomic region. Unfortunately, the drawback of a large window of informative tag markers is the necessity to explore a wide genomic region surrounding an associated SNP for a phenotypic trait. Great caution in experimental design should be applied for GWA studies involving disease since regions are likely not to be under selection. The LD estimates reported here can enhance the design of future GWA studies, especially in sample size and study design assessment, and the search for candidate genes.

## Supporting Information

Figure S1 **Position of the 1 Mb regions and spacing of the SNPs in 10 chromosomal regions of domestic cat.** a) Position of the 1 Mb regions in relation to centromere, mid-arm, and telomere. Black blocks represent centromere, except Chr. F2, which is an acrocentric chromosome, and red vertical lines represent the chosen 1 Mb region. b) Spacing of ∼ 150 SNPs in each of 10 chromosomal regions (A1, A2, B3, C2, D1, D2, D4, E2, F2, X, respectively) of 1 Mb length. SNPs are positioned to be denser at one end of each 1 Mb chromosomal region.
(TIFF)

Figure S2 **Variation in Tajima’s D estimates between domestic cat populations.** a) Tajima’s D estimate for each breed/population in each chromosomal region (see legend). b) Mean Tajima’s D estimate of values obtained for individual populations (a). c) Mean Tajima’s D estimate of values obtained for individual chromosomes. Error bars in (b) and (c) represent the standard deviation.
(TIFF)

Figure S3 **Effect of minor allele frequency choice on LD decay in domestic cat.** a) LD decay of Manx breed (n = 20). b) LD decay of Birman breed (n = 20). c) LD decay of Egyptian Mau Breed (n = 9). d) LD decay of Chartreux breed (n = 8). Regardless to the sample size, the LD decay appears to be inflated when using MAF of 0.1 due to the reduction in the number of pair-wise estimates of LD measure that are modeled to get a decay graph. Decay lines that do not show the point of 50% decay (solid circle or triangle) indicate that the 50% decay point is not reached at 1000 Kb. Decay lines that show abnormally an increase instead of decrease of *r^2^* as a function of distance such as in (b) is a result of small number of pair-wise comparisons.
(TIFF)

Figure S4 **Extent of LD in cat populations.** Bar plots represent the extent of LD for each population in each chromosomal region as well as the combined autosomal chromosomes and all chromosomes. The extent of LD represents the point of 50% decay of *r^2^* initial value. Instances where the extent of LD reaches 1000 Kb indicate that the decay of *r^2^* does not reach 50% of its initial value.
(TIFF)

Figure S5 **Haplotype structure and diversity of selected 10 chromosomal regions in domestic cat populations.** Haplotype analysis of chromosome regions (A1-X). Breeds/populations are represented on the y axis and the position of haplotype blocks are shown on the x axis. Haplotype blocks are represented by horizontal rectangles. The frequency of individual haplotypes within a block is proportional to the height of the color. Major haplotype is represented by black color, intermediate haplotypes with frequency >0.1 are represented by blue, light blue, red, green and yellow. Pink color represents the sum of the frequencies of all haploypes with a frequency <0.1. Empty regions between blocks indicate lack of any informative markers (MAF ≥0.05) and can be considered monomorphic across all individuals within a population.
(TIFF)

Figure S6 **Haplotype diversity of cat populations.** a) Haplotype diversity measured by the number of haplotype block defined in each chromosomal region. b) Haplotype diversity measured by the total number of haplotypes found in each chromosomal region. Legend corresponds to the ten chromosomal regions.
(TIFF)

Table S1 **Genomic and general summary of the 1** **Mb regions in cat LD analysis.**
(DOC)

Table S2 **GoldenGate assay SNP location, sequence, and genotypes of discovery cats.**
(XLS)

Table S3 **Population information and summary statistics.**
(DOC)

Table S4 **Distance (Kb) for achievement of 50% decay of the LD measure.** The (>1000) indicates that the LD measure did not reach 50% of its initial value at 1000 Kb.
(DOC)

Table S5 **Background means of cat LD estimates (** ***r^2^*** **) using MAF = 0.1.**
(DOC)

Table S6 **Fractions of pairs of SNPs with an** ***r^2^*** **value ≥0.8 at various distance classes.**
(DOC)
