# Supplementary figures and images for: Extent of Linkage Disequilibrium in the Domestic Cat, Felis silvestris catus, and Its Breeds
Source: PLoS One. 2013 Jan 7;8(1):e53537. doi: 10.1371/journal.pone.0053537 (PMC3538540; doi:10.1371/journal.pone.0053537)

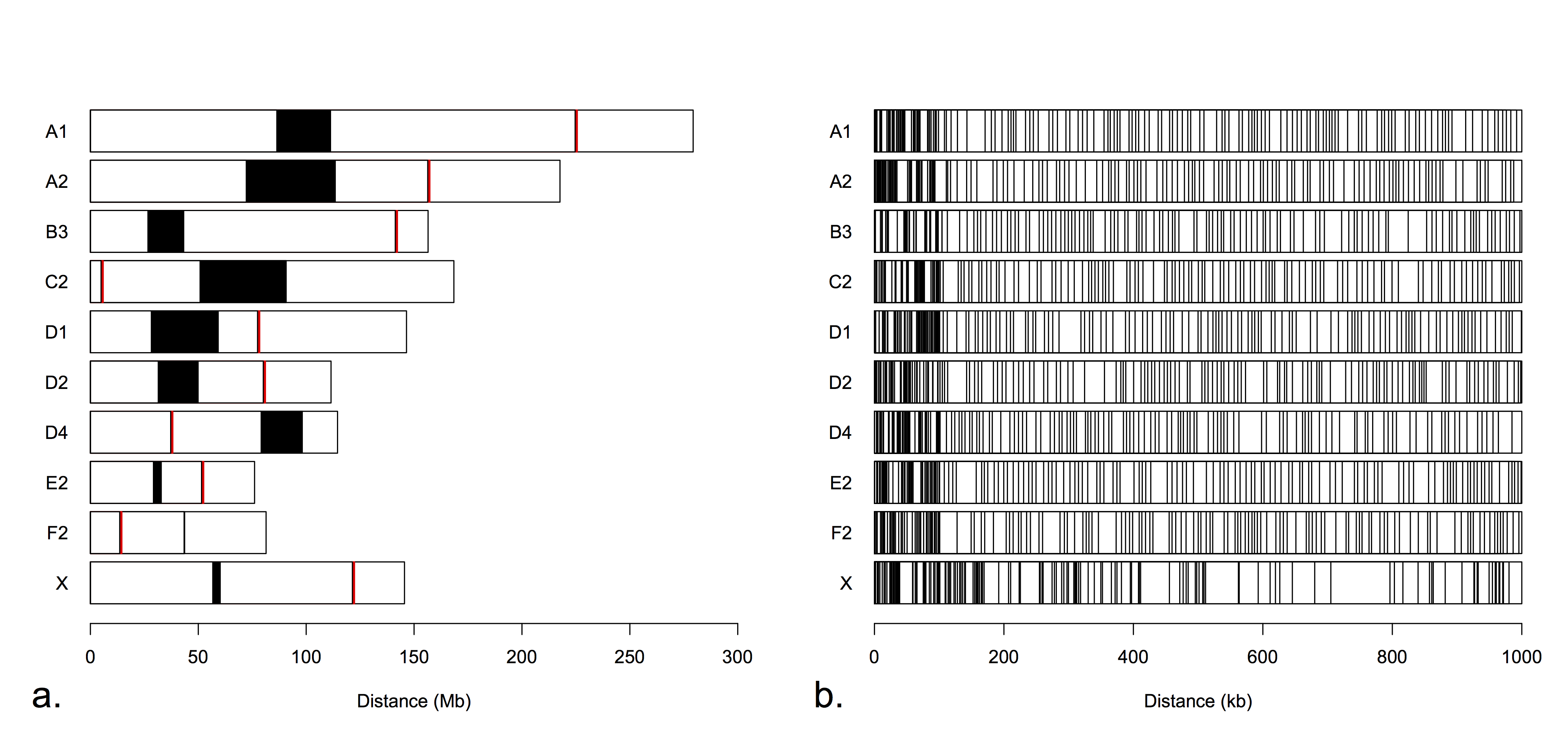

Supplement: Figure S1 — Position of the 1 Mb regions and spacing of the SNPs in 10 chromosomal regions of domestic cat. a) Position of the 1 Mb regions in relation to centromere, mid-arm, and telomere. Black blocks represent centromere, except Chr. F2, which is an acrocentric chromosome, and red vertical lines represent the chosen 1 Mb region. b) Spacing of ∼ 150 SNPs in each of 10 chromosomal regions (A1, A2, B3, C2, D1, D2, D4, E2, F2, X, respectively) of 1 Mb length. SNPs are positioned to be denser at one end of each 1 Mb chromosomal region. (TIFF) [file pone.0053537.s001.tiff]

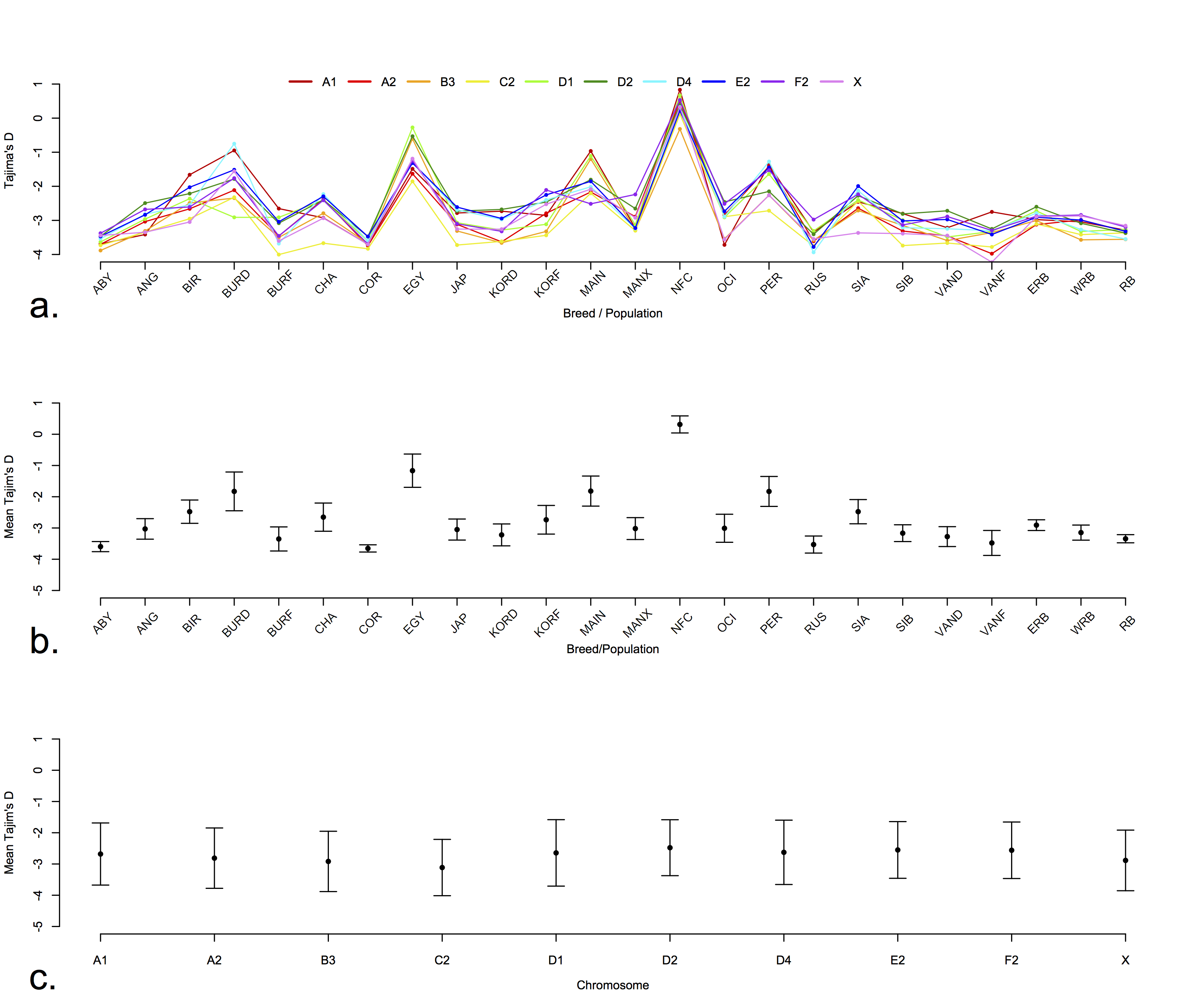

Supplement: Figure S2 — Variation in Tajima’s D estimates between domestic cat populations. a) Tajima’s D estimate for each breed/population in each chromosomal region (see legend). b) Mean Tajima’s D estimate of values obtained for individual populations (a). c) Mean Tajima’s D estimate of values obtained for individual chromosomes. Error bars in (b) and (c) represent the standard deviation. (TIFF) [file pone.0053537.s002.tiff]

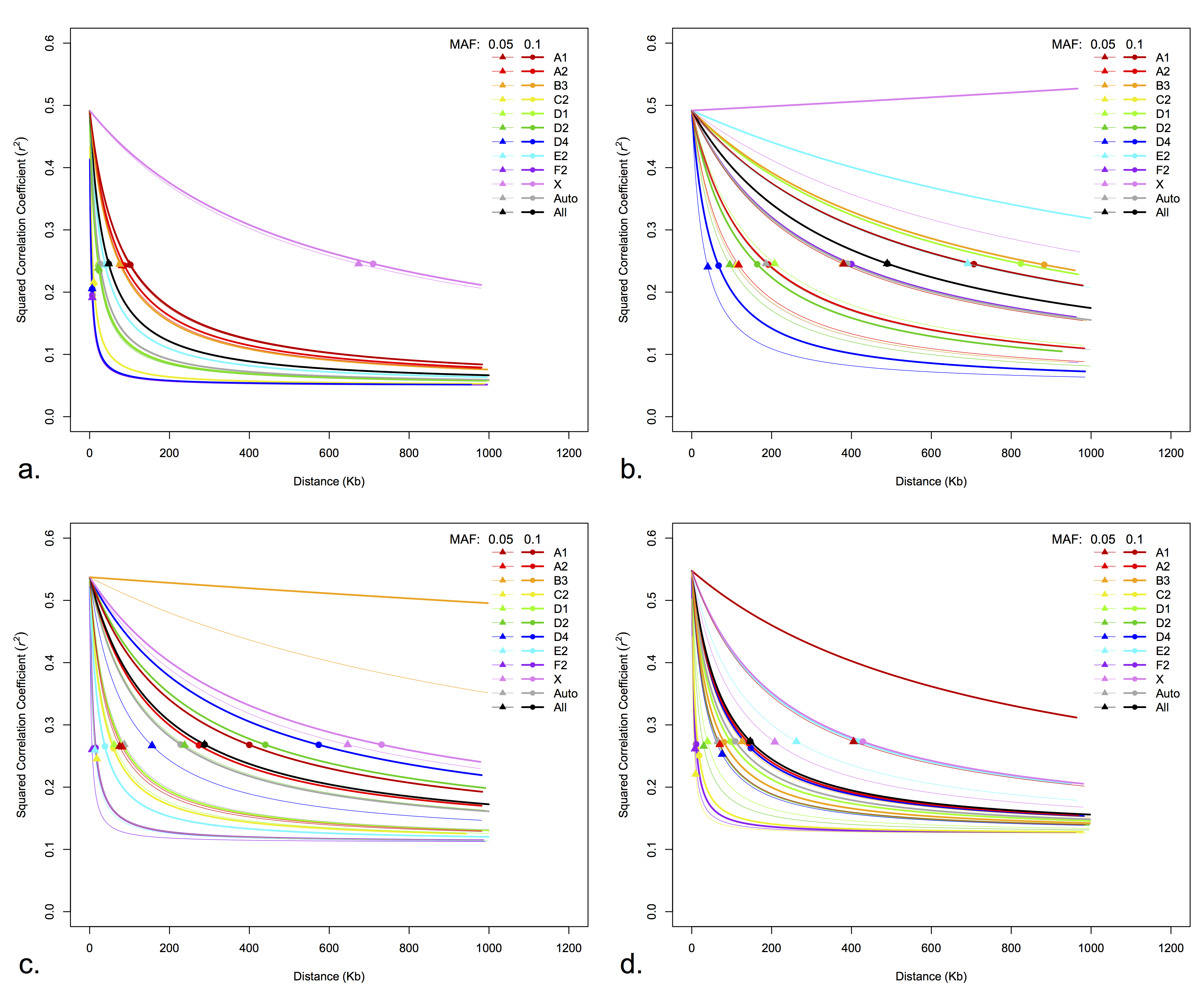

Supplement: Figure S3 — Effect of minor allele frequency choice on LD decay in domestic cat. a) LD decay of Manx breed (n = 20). b) LD decay of Birman breed (n = 20). c) LD decay of Egyptian Mau Breed (n = 9). d) LD decay of Chartreux breed (n = 8). Regardless to the sample size, the LD decay appears to be inflated when using MAF of 0.1 due to the reduction in the number of pair-wise estimates of LD measure that are modeled to get a decay graph. Decay lines that do not show the point of 50% decay (solid circle or triangle) indicate that the 50% decay point is not reached at 1000 Kb. Decay lines that show abnormally an increase instead of decrease of r2 as a function of distance such as in (b) is a result of small number of pair-wise comparisons. (TIFF) [file pone.0053537.s003.tiff]

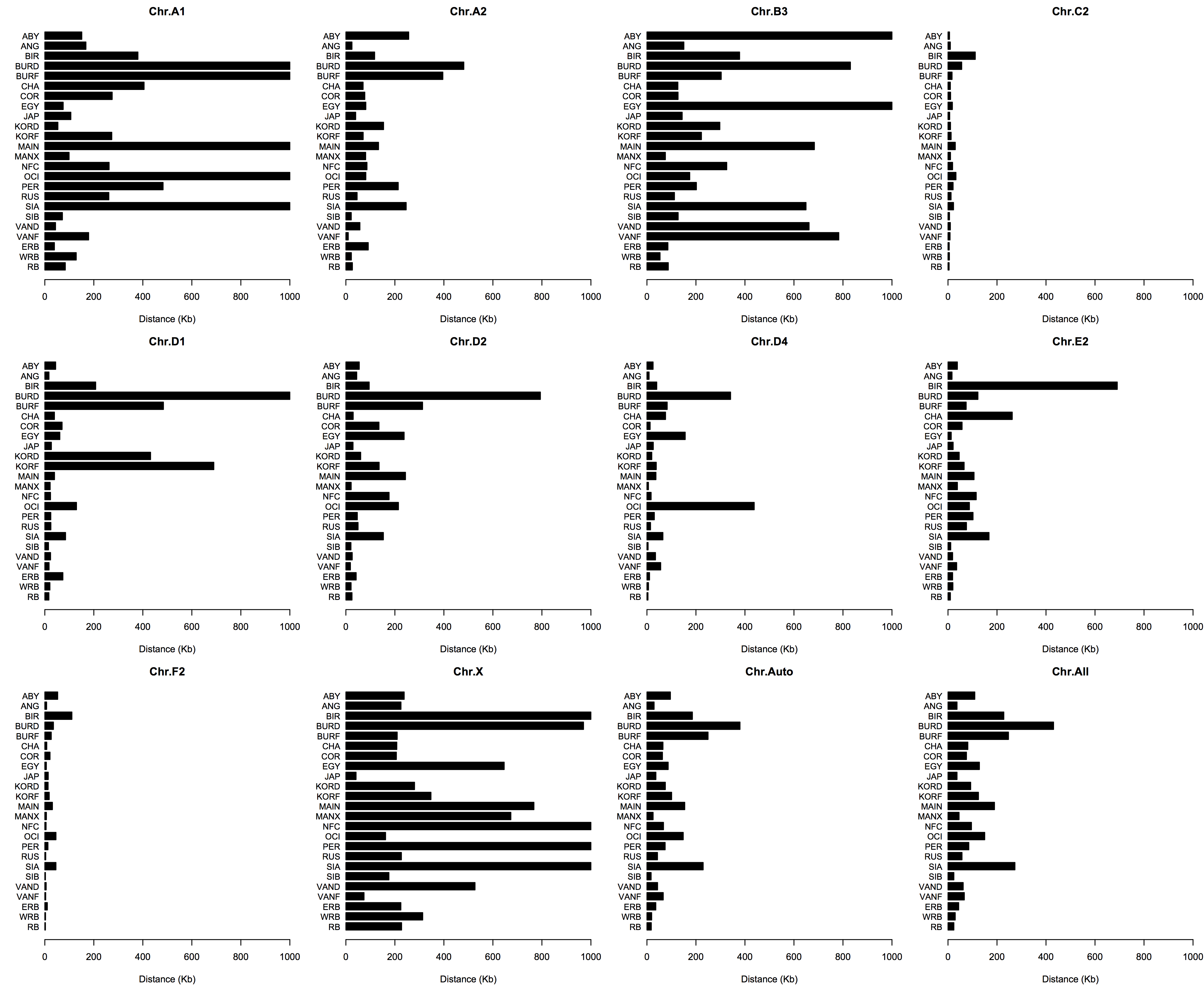

Supplement: Figure S4 — Extent of LD in cat populations. Bar plots represent the extent of LD for each population in each chromosomal region as well as the combined autosomal chromosomes and all chromosomes. The extent of LD represents the point of 50% decay of r2 initial value. Instances where the extent of LD reaches 1000 Kb indicate that the decay of r2 does not reach 50% of its initial value. (TIFF) [file pone.0053537.s004.tiff]

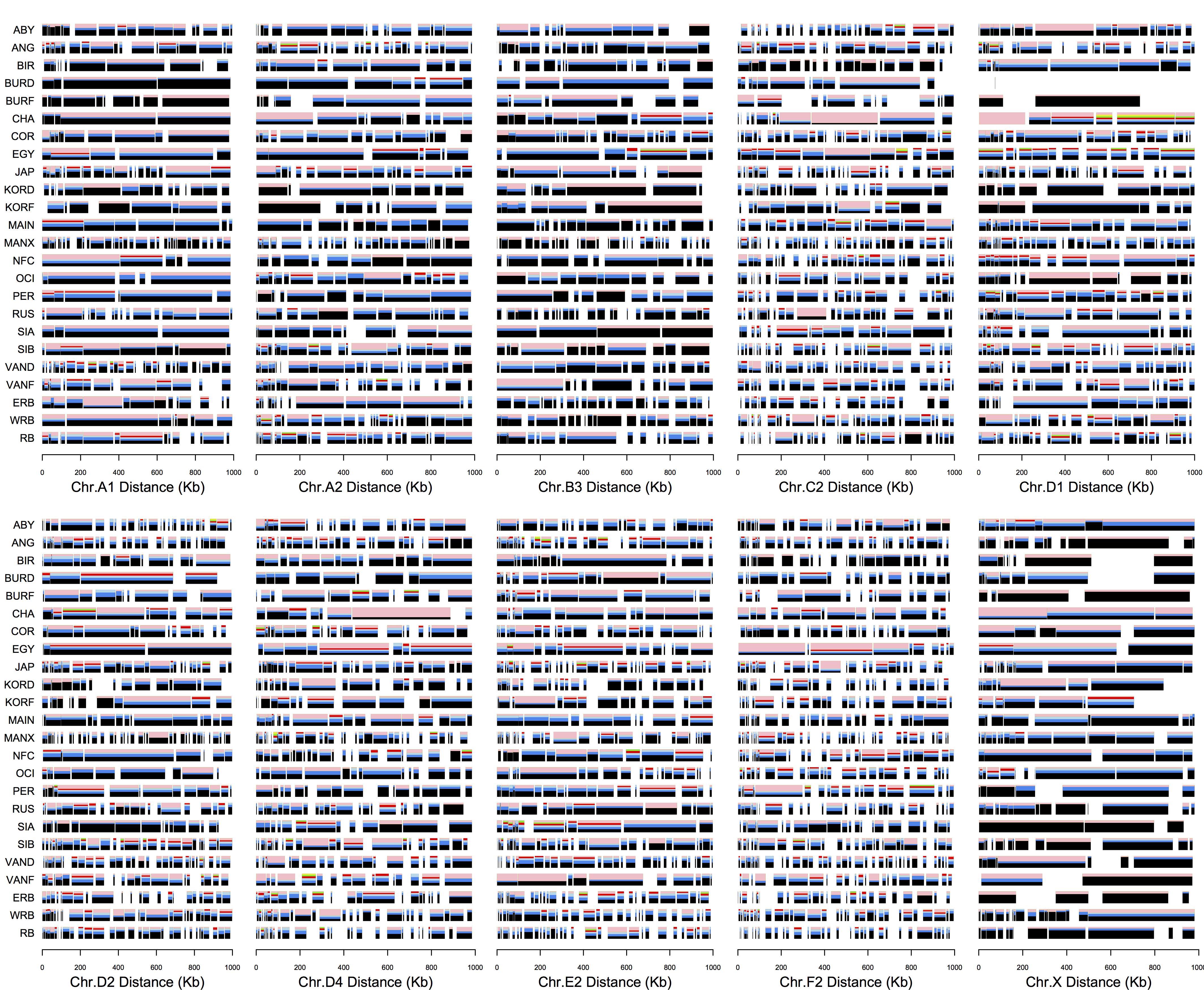

Supplement: Figure S5 — Haplotype structure and diversity of selected 10 chromosomal regions in domestic cat populations. Haplotype analysis of chromosome regions (A1-X). Breeds/populations are represented on the y axis and the position of haplotype blocks are shown on the x axis. Haplotype blocks are represented by horizontal rectangles. The frequency of individual haplotypes within a block is proportional to the height of the color. Major haplotype is represented by black color, intermediate haplotypes with frequency >0.1 are represented by blue, light blue, red, green and yellow. Pink color represents the sum of the frequencies of all haploypes with a frequency <0.1. Empty regions between blocks indicate lack of any informative markers (MAF ≥0.05) and can be considered monomorphic across all individuals within a population. (TIFF) [file pone.0053537.s005.tiff]

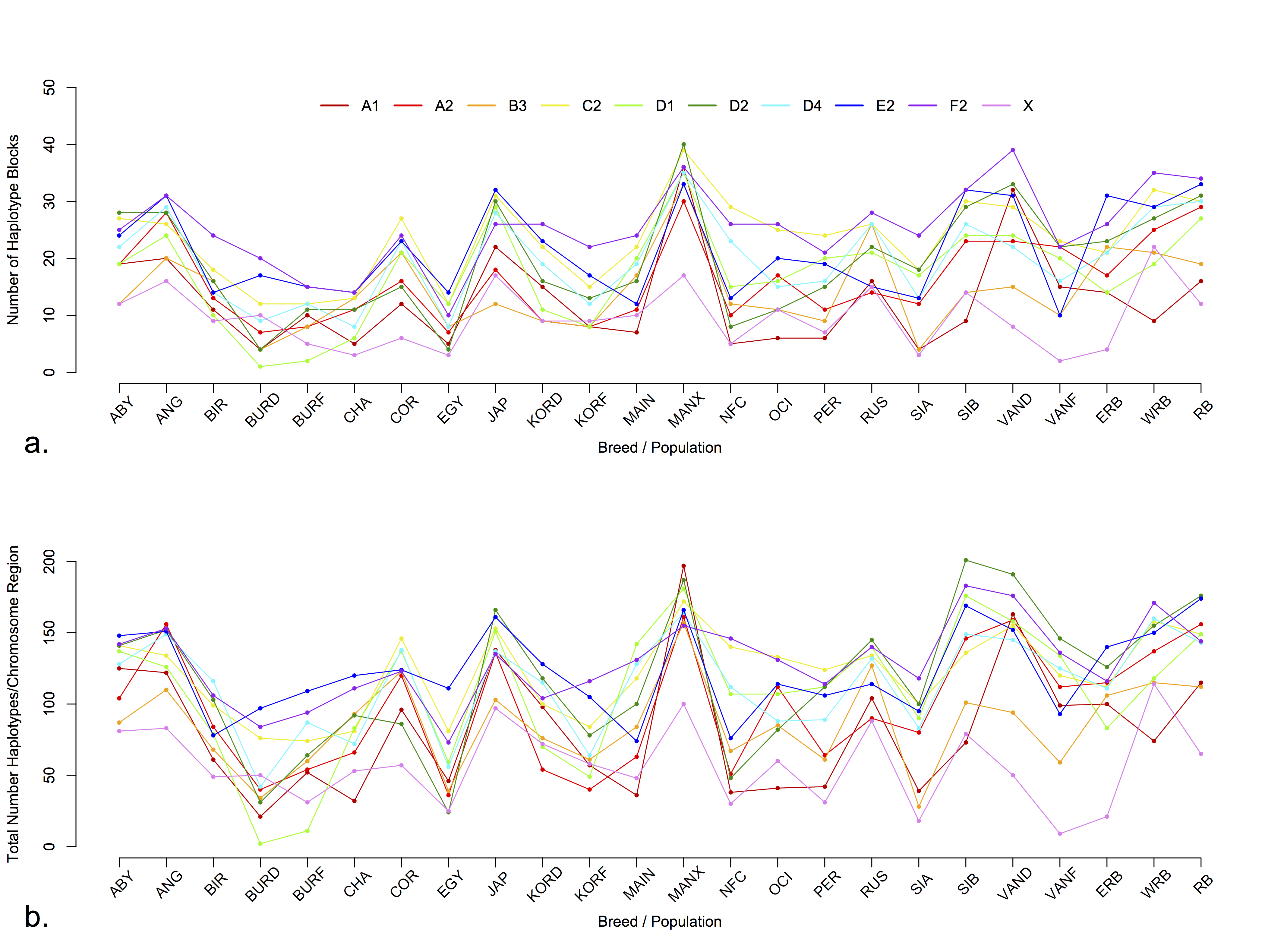

Supplement: Figure S6 — Haplotype diversity of cat populations. a) Haplotype diversity measured by the number of haplotype block defined in each chromosomal region. b) Haplotype diversity measured by the total number of haplotypes found in each chromosomal region. Legend corresponds to the ten chromosomal regions. (TIFF) [file pone.0053537.s006.tiff]
