# Supplementary material for: Extent of Linkage Disequilibrium in the Domestic Cat, Felis silvestris catus, and Its Breeds
Source: PLoS One. 2013 Jan 7;8(1):e53537. doi: 10.1371/journal.pone.0053537 (PMC3538540; doi:10.1371/journal.pone.0053537)
Supplement: Table S1 — Genomic and general summary of the 1 Mb regions in cat LD analysis. (DOC) [file pone.0053537.s007.doc]

Table S1: Genomic and general summary of the 1Mb regions in cat LD analysis.

| **Chr** | **No. SNPs** | | | **Position new assembly** | | | **felCat4 assembly** | | | | | |
| --- | --- | --- | --- | --- | --- | --- | --- | --- | --- | --- | --- | --- |
|  | **Total** | **Used** | **Failed** | **Start** | **End** | **Length (bp)** | **Start** | **End** | **Length (bp)** | **%GC** | **No.**  **Gene*** | **No. Repeats**** |
| A1 | 154 | 151 | 3 | 224644927 | 225641786 | 996,859 | 219018905 | 219876776 | 857,872 | 43.8 | 0 | 86 |
| A2 | 155 | 147 | 8 | 156328646 | 157321810 | 993,164 | 154175441 | 155043070 | 867,630 | 37.3 | 13 | 92 |
| B3 | 153 | 148 | 5 | 141292628 | 142289729 | 997,101 | 131438290 | 132349753 | 911,464 | 37.4 | 9 | 82 |
| C2 | 154 | 142 | 12 | 4945241 | 5941481 | 996,240 | 5066239 | 6036783 | 970,545 | 49.3 | 20 | 136 |
| D1 | 154 | 151 | 3 | 77464646 | 78463979 | 999,333 | 75576122 | 76476050 | 899,929 | 36.7 | 6 | 109 |
| D2 | 152 | 148 | 4 | 80117174 | 81115518 | 998,344 | 77871061 | 78771280 | 900,220 | 39.1 | 8 | 81 |
| D4 | 153 | 147 | 6 | 37213961 | 38198807 | 984,846 | 36634725 | 37581020 | 946,296 | 40.1 | 17 | 95 |
| E2 | 154 | 147 | 7 | 51512246 | 52511185 | 998,939 | 51510648 | 52448199 | 937,552 | 41.6 | 7 | 128 |
| F2 | 154 | 142 | 12 | 13591589 | 14587072 | 995,483 | 9987235*** | 9216856 | 770,380 | 43.0 | 10 | 109 |
| X | 153 | 140 | 13 | 121417485 | 122397750 | 980,265 | 112690481 | 113689635 | 999,155 | 42.0 | 14 | 106 |
| **Tot** | **1536** | **1463** | **73** |  |  |  |  |  |  |  |  |  |
| **%** |  | **95.1** | **4.99** |  |  |  |  |  |  |  |  |  |

* Number of coding human genes in each region.

** Number of simple repeats of > 10 repeat units in each region.

*** The region in the felCat4 assembly appears in opposite orientation.

The genomic analysis was conducted on the felCat4 assembly since the new assembly has not yet been released.
