# Supplementary material for: Extent of Linkage Disequilibrium in the Domestic Cat, Felis silvestris catus, and Its Breeds
Source: PLoS One. 2013 Jan 7;8(1):e53537. doi: 10.1371/journal.pone.0053537 (PMC3538540; doi:10.1371/journal.pone.0053537)
Supplement: Table S3 — Population information and summary statistics. (DOC) [file pone.0053537.s009.doc]

Table S3: Population information and summary statistics.

| **Breed/Random bred** | **Sample source *** | | | **Percent %** | | | **Mean** | | | | | |
| --- | --- | --- | --- | --- | --- | --- | --- | --- | --- | --- | --- | --- |
|  | **DNA** | **WGA** | **t-test (*p*)** | **Mono** | **MAF <0.05** | **Call rate < 80%** | **H_e_** | **H_o_** | **F_is_** | **Block No.**** | **Hap No.***** | **Tajima’s D ****** |
| Abyssinian | 0 | 21 | - | 13.7 | 12.9 | 8.2 | 0.262 | 0.255 | -0.024 | 20.7 | 123.4 | -3.60 |
| Turkish Angora | 2 | 12 | - | 4.9 | 14.6 | 12.6 | 0.289 | 0.277 | -0.040 | 25.3 | 133.6 | -3.03 |
| Birman | 5 | 15 | 0.26 | 20.4 | 11.8 | 0.6 | 0.219 | 0.213 | -0.022 | 14.5 | 84.3 | -2.48 |
| Burmese (Domestic) | 6 | 13 | 0.30 | 40.3 | 7.2 | 2.3 | 0.192 | 0.187 | -0.017 | 8.8 | 47.7 | -1.83 |
| Burmese (Foreign) | 0 | 19 | - | 40.7 | 11.6 | 9.0 | 0.171 | 0.166 | -0.018 | 9.8 | 63.6 | -3.35 |
| Chartreux | 2 | 6 | - | 26.3 | 0.0 | 10.0 | 0.265 | 0.246 | -0.055 | 9.8 | 80.3 | -2.65 |
| Cornish Rex | 4 | 17 | 0.02 | 15.7 | 8.3 | 6.6 | 0.269 | 0.262 | -0.023 | 18.8 | 115 | -3.65 |
| Egyptian Mau | 4 | 5 | 0.94 | 28.0 | 0.0 | 4.5 | 0.254 | 0.239 | -0.045 | 8.3 | 55 | -1.17 |
| Japanese Bobtail | 0 | 12 | - | 4.6 | 14.0 | 11.7 | 0.304 | 0.290 | -0.047 | 24.5 | 137.7 | -3.05 |
| Korat (Domestic) | 6 | 13 | 8e-5 | 30.9 | 10.4 | 6.8 | 0.197 | 0.191 | -0.021 | 15.9 | 93.5 | -3.22 |
| Korat (Foreign) | 11 | 6 | 0.28 | 38.9 | 9.2 | 3.3 | 0.189 | 0.182 | -0.020 | 12 | 71.2 | -2.74 |
| Maine Coon | 0 | 19 | - | 20.2 | 6.2 | 0.9 | 0.246 | 0.239 | -0.023 | 15.8 | 92.4 | -1.82 |
| Manx | 11 | 9 | 0.77 | 2.5 | 6.8 | 1.8 | 0.303 | 0.294 | -0.027 | 33.4 | 164 | -3.02 |
| Norwegian Forest Cat | 0 | 20 | - | 14.9 | 11.8 | 1.0 | 0.258 | 0.251 | -0.023 | 14.6 | 81.5 | 0.31 |
| Ocicat | 9 | 12 | 0.02 | 18.7 | 10.9 | 4.4 | 0.262 | 0.255 | -0.022 | 15.8 | 95.3 | -3.01 |
| Persian | 7 | 12 | 0.60 | 22.4 | 9.5 | 1.8 | 0.236 | 0.229 | -0.022 | 14.8 | 85.5 | -1.83 |
| Russian Blue | 4 | 14 | 0.06 | 13.5 | 10.9 | 9.0 | 0.260 | 0.252 | -0.028 | 20.9 | 121.4 | -3.53 |
| Siamese | 7 | 12 | 0.09 | 31.6 | 8.9 | 2.5 | 0.204 | 0.199 | -0.020 | 12.6 | 75.1 | -2.48 |
| Siberian | 5 | 14 | 0.31 | 9.1 | 6.3 | 5.8 | 0.278 | 0.270 | -0.027 | 23.3 | 141.3 | -3.17 |
| Turkish Van (Domestic) | 4 | 15 | 0.03 | 4.4 | 9.8 | 10.6 | 0.297 | 0.288 | -0.029 | 25.6 | 144.3 | -3.28 |
| Turkish Van (Foreign) | 2 | 10 | - | 24.1 | 9.5 | 12.8 | 0.247 | 0.235 | -0.038 | 16.2 | 103.3 | -3.48 |
| Eastern Random Bred | 0 | 22 | - | 22.1 | 13.0 | 3.7 | 0.233 | 0.227 | -0.020 | 19.3 | 103 | -2.91 |
| Western Random Bred | 8 | 13 | 5e-5 | 5.0 | 7.5 | 3.3 | 0.291 | 0.284 | -0.025 | 24.8 | 135.2 | -3.15 |
| Random Bred | - | - | - | 2.1 | 14.0 | 2.5 | 0.289 | 0.286 | -0.012 | 26.1 | 138.3 | -3.34 |
| **Total** | **97** | **311** | **Avg.** | **19.0** | **9.4** | **5.7** | **0.251** | **0.242** | **-0.027** | **17.98** | **103.58** | **-2.73** |

* A t-test performed on the number of heterozygous genotype calls in samples of DNA and WGA sources. Each population has been done independently. A two–way ANOVA accounting for population and sample source showed statistically not significant differences in heterozygous calls (*p* = 0.136).

** Mean Number of haplotype blocks across the ten chromosomal regions.

*** Mean number of total haplotypes across all regions.

**** Mean Tajima’s D across all regions.

DNA: direct DNA source, WGA: Whole-genome amplified sample, Mono: monomorphic SNPs, MAF: minor allele frequency, H_e_: expected heterozygosity, H_o_: observed heterozygosity, F_is_: inbreeding coefficient.
