# Supplementary material for: Extent of Linkage Disequilibrium in the Domestic Cat, Felis silvestris catus, and Its Breeds
Source: PLoS One. 2013 Jan 7;8(1):e53537. doi: 10.1371/journal.pone.0053537 (PMC3538540; doi:10.1371/journal.pone.0053537)
Supplement: Table S4 — Distance (Kb) for achievement of 50% decay of the LD measure. The (>1000) indicates that the LD measure did not reach 50% of its initial value at 1000 Kb. (DOC) [file pone.0053537.s010.doc]

Table S4: Distance (Kb) for achievement of 50% decay of the LD measure. The (>1000) indicates that the LD measure did not reach 50% of its initial value at 1000Kb.

| **Popn.** | **Chromosome** | | | | | | | | | | | |
| --- | --- | --- | --- | --- | --- | --- | --- | --- | --- | --- | --- | --- |
|  | **A1** | **A2** | **B3** | **C2** | **D1** | **D2** | **D4** | **E2** | **F2** | **X** | **Auto** | **All** |
| **ABY** | 151 | 257 | >1000 | 6 | 44 | 55 | 25 | 38 | 53 | 238 | 96 | 109 |
| **ANG** | 168 | 25 | 151 | 10 | 17 | 44 | 9 | 17 | 7 | 225 | 29 | 37 |
| **BIR** | 380 | 117 | 378 | 111 | 208 | 95 | 40 | 691 | 111 | >1000 | 186 | 228 |
| **BURD** | >1000 | 481 | 831 | 56 | >1000 | 794 | 341 | 122 | 35 | 970 | 380 | 431 |
| **BURF** | >1000 | 396 | 303 | 17 | 484 | 313 | 83 | 74 | 26 | 210 | 249 | 247 |
| **CHA** | 405 | 70 | 126 | 10 | 39 | 30 | 76 | 262 | 8 | 208 | 66 | 81 |
| **COR** | 275 | 77 | 127 | 11 | 71 | 135 | 13 | 58 | 21 | 206 | 63 | 75 |
| **EGY** | 75 | 82 | >1000 | 18 | 61 | 238 | 156 | 13 | 6 | 646 | 87 | 128 |
| **JAP** | 106 | 40 | 144 | 7 | 27 | 29 | 26 | 21 | 14 | 42 | 37 | 37 |
| **KORD** | 53 | 154 | 298 | 10 | 432 | 61 | 20 | 46 | 13 | 280 | 75 | 93 |
| **KORF** | 273 | 71 | 223 | 13 | 690 | 136 | 38 | 66 | 18 | 347 | 101 | 124 |
| **MAIN** | >1000 | 133 | 683 | 30 | 40 | 243 | 37 | 106 | 31 | 768 | 154 | 190 |
| **MANX** | 99 | 81 | 75 | 10 | 22 | 22 | 6 | 38 | 7 | 673 | 25 | 45 |
| **NFC** | 262 | 86 | 326 | 19 | 23 | 176 | 17 | 115 | 5 | >1000 | 68 | 96 |
| **OCI** | >1000 | 82 | 175 | 33 | 129 | 215 | 438 | 87 | 46 | 162 | 148 | 150 |
| **PER** | 483 | 214 | 202 | 21 | 25 | 47 | 30 | 102 | 13 | >1000 | 74 | 85 |
| **RUS** | 261 | 46 | 112 | 13 | 25 | 50 | 15 | 76 | 4 | 227 | 43 | 57 |
| **SIA** | >1000 | 246 | 649 | 23 | 85 | 153 | 65 | 168 | 46 | >1000 | 230 | 273 |
| **SIB** | 72 | 22 | 128 | 6 | 15 | 21 | 5 | 11 | 3 | 176 | 17 | 24 |
| **VAND** | 44 | 57 | 662 | 10 | 24 | 26 | 35 | 19 | 6 | 527 | 44 | 62 |
| **VANF** | 179 | 10 | 783 | 9 | 17 | 19 | 56 | 36 | 4 | 74 | 67 | 67 |
| **ERB** | 39 | 91 | 86 | 5 | 74 | 42 | 11 | 19 | 10 | 225 | 36 | 44 |
| **WRB** | 128 | 23 | 54 | 6 | 21 | 21 | 6 | 20 | 3 | 314 | 19 | 30 |
| **RB** | 84 | 27 | 87 | 5 | 17 | 25 | 4 | 10 | 3 | 227 | 18 | 24 |
