# Supplementary material for: Extent of Linkage Disequilibrium in the Domestic Cat, Felis silvestris catus, and Its Breeds
Source: PLoS One. 2013 Jan 7;8(1):e53537. doi: 10.1371/journal.pone.0053537 (PMC3538540; doi:10.1371/journal.pone.0053537)
Supplement: Table S5 — Background means of cat LD estimates ( r2 ) using MAF = 0.1. (DOC) [file pone.0053537.s011.doc]

**Table S5: Background means of cat LD estimates (*r^2^***) using MAF = 0.1.

| **Breeds** | **No. of pair-wise comparisons** | ***r^2^*** |
| --- | --- | --- |
| ABY | 274700 | 0.06 |
| ANG | 322000 | 0.09 |
| BIR | 242900 | 0.06 |
| BURD | 188500 | 0.06 |
| BURF | 89720 | 0.06 |
| CHA | 253400 | 0.16 |
| COR | 302800 | 0.06 |
| EGY | 297800 | 0.11 |
| ERB | 236500 | 0.05 |
| JAP | 321000 | 0.11 |
| KORD | 146600 | 0.06 |
| KORF | 139000 | 0.06 |
| MAIN | 301200 | 0.06 |
| MANX | 495700 | 0.06 |
| NFC | 327700 | 0.05 |
| OCI | 288700 | 0.05 |
| PER | 266400 | 0.06 |
| RB | 422300 | 0.04 |
| RUS | 247500 | 0.06 |
| SIA | 181100 | 0.07 |
| SIB | 370000 | 0.06 |
| VAND | 366500 | 0.07 |
| VANF | 178300 | 0.09 |
| WRB | 422000 | 0.06 |
| **Average** | **278430** | **0.07** |
