# Supplementary material for: Extent of Linkage Disequilibrium in the Domestic Cat, Felis silvestris catus, and Its Breeds
Source: PLoS One. 2013 Jan 7;8(1):e53537. doi: 10.1371/journal.pone.0053537 (PMC3538540; doi:10.1371/journal.pone.0053537)
Supplement: Table S6 — Fractions of pairs of SNPs with an r2 value ≥0.8 at various distance classes. (DOC) [file pone.0053537.s012.doc]

**Table S6: Fractions of pairs of SNPs with an *r^2^*** value ≥ 0.8 at various distance classes.

| **Breed** | **Distance between pairs of markers (Kb)** | | | | | | | | | |
| --- | --- | --- | --- | --- | --- | --- | --- | --- | --- | --- |
|  | **0-20** | **20-40** | **40-60** | **60-80** | **80-100** | **100-200** | **200-400** | **400-600** | **600-800** | **800-1000** |
| ABY | 0.08 | 0.05 | 0.05 | 0.06 | 0.05 | 0.03 | 0.02 | 0.02 | 0.01 | 0.00 |
| ANG | 0.05 | 0.03 | 0.02 | 0.02 | 0.02 | 0.02 | 0.01 | 0.01 | 0.00 | 0.00 |
| BIR | 0.13 | 0.10 | 0.10 | 0.08 | 0.09 | 0.07 | 0.05 | 0.03 | 0.02 | 0.02 |
| BURD | 0.29 | 0.21 | 0.19 | 0.16 | 0.18 | 0.15 | 0.10 | 0.05 | 0.03 | 0.05 |
| BURF | 0.12 | 0.10 | 0.07 | 0.07 | 0.08 | 0.05 | 0.04 | 0.03 | 0.03 | 0.03 |
| CHA | 0.12 | 0.07 | 0.09 | 0.07 | 0.06 | 0.06 | 0.05 | 0.05 | 0.03 | 0.02 |
| COR | 0.07 | 0.04 | 0.04 | 0.03 | 0.03 | 0.02 | 0.02 | 0.01 | 0.01 | 0.01 |
| EGY | 0.15 | 0.10 | 0.12 | 0.09 | 0.09 | 0.08 | 0.06 | 0.04 | 0.03 | 0.03 |
| JAP | 0.04 | 0.03 | 0.02 | 0.02 | 0.02 | 0.01 | 0.01 | 0.01 | 0.01 | 0.01 |
| KORD | 0.09 | 0.07 | 0.05 | 0.04 | 0.04 | 0.04 | 0.03 | 0.01 | 0.01 | 0.01 |
| KORF | 0.12 | 0.09 | 0.08 | 0.07 | 0.05 | 0.05 | 0.03 | 0.02 | 0.02 | 0.02 |
| MAIN | 0.19 | 0.14 | 0.14 | 0.11 | 0.11 | 0.08 | 0.05 | 0.03 | 0.02 | 0.01 |
| MANX | 0.05 | 0.04 | 0.03 | 0.03 | 0.03 | 0.02 | 0.01 | 0.00 | 0.00 | 0.00 |
| NFC | 0.21 | 0.14 | 0.13 | 0.10 | 0.09 | 0.07 | 0.04 | 0.02 | 0.02 | 0.02 |
| OCI | 0.12 | 0.08 | 0.08 | 0.06 | 0.06 | 0.04 | 0.03 | 0.02 | 0.02 | 0.01 |
| PER | 0.15 | 0.10 | 0.10 | 0.08 | 0.08 | 0.04 | 0.02 | 0.01 | 0.01 | 0.00 |
| RUS | 0.05 | 0.04 | 0.04 | 0.03 | 0.03 | 0.03 | 0.02 | 0.01 | 0.01 | 0.00 |
| SIA | 0.16 | 0.12 | 0.13 | 0.10 | 0.10 | 0.10 | 0.07 | 0.05 | 0.02 | 0.01 |
| SIB | 0.04 | 0.03 | 0.03 | 0.02 | 0.02 | 0.01 | 0.01 | 0.00 | 0.00 | 0.00 |
| VAND | 0.06 | 0.04 | 0.04 | 0.05 | 0.04 | 0.03 | 0.02 | 0.01 | 0.01 | 0.01 |
| VANF | 0.05 | 0.06 | 0.05 | 0.04 | 0.05 | 0.03 | 0.03 | 0.03 | 0.01 | 0.01 |
| ERB | 0.08 | 0.06 | 0.03 | 0.03 | 0.04 | 0.02 | 0.01 | 0.00 | 0.00 | 0.00 |
| WRB | 0.06 | 0.03 | 0.03 | 0.02 | 0.02 | 0.02 | 0.01 | 0.00 | 0.00 | 0.00 |
| RB | 0.05 | 0.02 | 0.02 | 0.01 | 0.01 | 0.01 | 0.01 | 0.00 | 0.00 | 0.00 |
